# Various Color Light Emission from Single, Double, and Triple Eu^**3+**^/Tb^**3+**^/Tm^**3+**^ Doped Borate Glass Excited by UV Light

**DOI:** 10.1007/s10895-023-03502-x

**Published:** 2023-12-05

**Authors:** Ismail Kashif, Asmaa Ratep

**Affiliations:** 1 Faculty of Science, Department of Physics, Al-Azhar University, Nasr City, Cairo Egypt; 2 Faculty of Women for Art, Science & Education, Department of Physics, Ain Shams University, Heliopolis, Cairo Egypt

**Keywords:** Oxide glass doped with single, double, or triple Tm3+, Tb3+, and Eu3+ ions, Optical properties (absorption, excitation, and emission), CIE color chromaticity

## Abstract

Lithium-aluminum-borate glasses doped with single, double, or triple Tm^3+^, Tb^3+^, or Eu^3+^ ions, respectively, at several concentrations were prepared. Structural characterization was performed using optical absorption and luminescence spectroscopy. The transition for the rare earth ions under study was observed in optical absorption and some for-host glass at wavelengths less than 300 nm. The luminescence of Eu^3+^, Tb^3+^, and Tm^3+^ is presented as bright red, green, and blue emissions, respectively. White light was produced by adjusting the excitation wavelength and doping concentration of Tm_2_O_3_, Eu_2_O_3_, and Tb_4_O_7_. The sample doped with triple rare earth ions produced color emissions from Tb and Tm to Eu, which varied in hue based on the excitation wavelengths of 350, 360, and 370 nm. The glass samples under investigation may be promising for optoelectronic devices and security applications such as data encryption.

## Introduction

Researchers have shown significant interest in glass samples doped with rare-earth ions for various applications. Such as color displays, a developing field in medical devices, high-density optical data storage and reading, laser optical fibers, optical amplifiers, and visible display devices [1–7]. Owing to the internal shielding of electrons by the 5 s and 5p shells, the strong transitions within the 4fN of the rare-earth ions that cause it to occur are not influenced by any external fields [8, 9]. According to research on various glass systems that serve as hosts for rare earth ions [10–16], borate glass is a promising material owing to its high transparency, broad range of formation, thermal and mechanical stability, and high solubility of lanthanum ions. Using the melt-quenching process, single- and double-doped (Tb^**3+**^ and Eu^**3+**^) lithium borate glass was created, and the photoluminescence properties of the glass samples were investigated. The CIE values change from warm red to cool white [17]. For the first time, borate glass doped with Ce^**3+**^, Tb^**3+**^, and Mn^**2+**^ produced white light when excited by UV light [18]. It took time and effort to develop a single-host glass system that emitted white light. Lin et al. [19] found that SiO_**2**_-Al_**2**_O_**3**_-CaO-CaF_**2**_ glass was excited by ultraviolet light and doped with Tb/Sm. Furthermore, Zhu et al. [20] produced Tm/Tb/Sm silicate glass at wavelengths below those of the near-ultraviolet excitation. However, it failed to provide the expected outcomes.

The melt quenching technique was used to produce the glass samples CaO-Al_**2**_O_**3**_-B_**2**_O_**3**_-RE_**2**_O_**3**_ (RE = Eu, Tb, and Tm). The photoluminescence of these glass samples was investigated, and mixed bands of blue, green, and reddish-orange light were observed in their emission spectra [21]. Samples under 360 nm excitation emit white light. In addition, the energy transfer (ET) between Tb^**3+**^ and Eu^**3+**^ ions in triply doped glasses were validated. Hence, glass samples are potential candidates for white LEDs.

The characteristics of the glass samples were susceptible to variations in the Tm_2_O_3_ concentration. According to the findings of this study, glass samples may be beneficial for laser and communication applications [22].

The goal was to prepare samples for a glass system that produces white light by mixing an appropriate combination of blue, green, and red emissions. Tm_**2**_O_**3**_, Tb_**4**_O_**7**_, and Eu_**2**_O_**3**_ were anesthetized in one glass group. By adding, first, each element separately, then, second, two elements together, and finally, all three elements are combined. A series of glass samples containing one rare earth ion (Tm or Tb or Eu), two rare earth ions (Tm + Tb or Tm + Eu or Tb + Eu), or three rare earth ions (Tm + Tb + Eu) were prepared. The photoluminescence behavior of the single-, double-, and triple-doped glass samples was examined. We analyzed and investigated various lanthanide energy transfer processes that occur in various doped glass samples. The effect of adding an equal amount of double and triple rare earth ions on photoluminescence spectra (PL) was also studied. It also shows how the intensity of the excitation energy can control the color change of radiation emitted by the sample.

## Experimental Work

Glass samples were prepared according to Table 1 composition. To remove the hydrogen and carbon contained in the raw materials, the samples were held for 30 min at 400 °C and then melted at 1000 °C for an hour. A high-temperature liquid (1000 °C) was poured between the two copper plates at room temperature (quenching melt technique).

**Table 1:** Glass sample compositions

Li_**2**_O	Al_**2**_O_**3**_	B_**2**_O_**3**_	Tm_**2**_O_**3**_	Tb_**4**_O_**7**_	Eu_**2**_O_**3**_
25	5	70	1	0	0
25	5	70	0	1	0
25	5	70	0	0	1
25	5	70	0.5	0.5	0
25	5	70	0	0.5	0.5
25	5	70	0.5	0	0.5
25	5	70	0.33	0.33	0.33

A Philips Analytical X-ray diffraction system, type PW3710, based on a Cu tube anode with wavelengths of Kα1 = 1.54060 Å and Kα2 = 1.54439°A, was used to confirm the glassy samples. The generator current was 30 mA, the generator tension was 40 kV. 10^o^ was the start angle (2θ) and 70^o^ was the end angle. To measure the optical transmission spectra, a computerized recording spectrophotometer was used to measure the wavelengths between 190 and 2500 nm (JASCO, V-570). Room-temperature emission spectra were obtained using a 150 W Xenon arc lamp and a JASCO FP-8300 spectrofluorometer.

## Results and Discussion

**Fig. 1:**
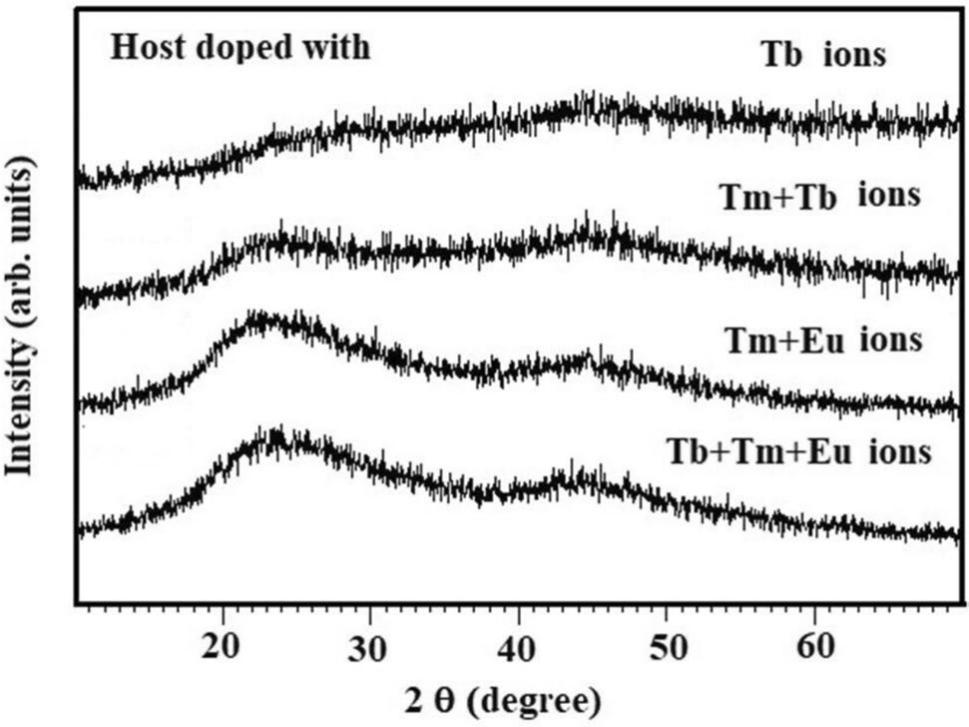
The X-ray diffraction patterns of the samples

Figure 1 shows the X-ray diffraction patterns of the studied samples. The two broad peaks at 2θ equal 23°and 43°are the diffracted X-ray peaks from the amorphous network of the rare-earth-doped glass. The broad diffraction peaks indicate disordered long-range order arrangements in the amorphous state of the studied samples. An X-ray diffraction pattern with a broader peak may signify a smaller crystal, a fault in the crystalline structure, or even an amorphous sample that lacks perfect crystalline. The Scherrer equation indicates that increased broadening will be caused by lower crystal sizes. Peak width and crystal size are inversely correlated. A thinner peak is related to a larger crystal. A broader peak indicates the presence of a smaller crystal or an amorphous material.

**Fig. 2:**
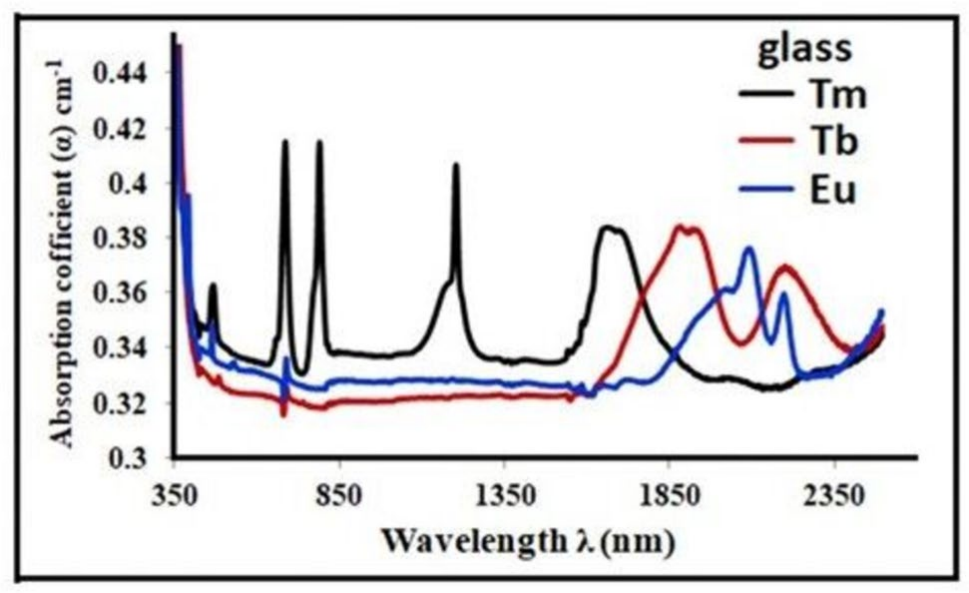
The absorption spectra of glass samples doped with various RE ions

Figure 2 shows the absorption spectra of the glass samples doped with various RE ions. Figure 2 shows the transition in the RE brought about by 4f-4f as well as the lower absorption and high transmission anticipated at a certain wavelength. The transition that evolved in the host glass may be the cause of the absorption peaks below 350 nm [23]. The transition from the ground state ^**3**^H_**6**_ to several excited states is visible in the glass sample doped with Tm_**2**_O_**3**_ [24, 25]. At 350, 456, 686, 790, 1202, and 1648 nm, for ^**1**^D_**2**_, ^**1**^G_**4**_, ^**3**^F_**3**_ + ^**3**^F_**2**_, ^**3**^H_**4**_, ^**3**^H_**5**_, and ^**3**^F_**4**_ are the corresponding wavelengths. Broad peaks in the near-IR region, which correspond to the ^**7**^F_**6**_ → ^**7**^F_**0**_, ^**7**^F_**1**_, and ^**7**^F_**2**_ transitions at 1906, 1870, and 1756 nm and ^**7**^F_**6**_ → ^**7**^F_**3**_ at 2174 nm, are visible in the sample doped with Tb_**4**_O_**7**_ [26–28]. However, the sample doped with Eu_**2**_O_**3**_ exhibits a transition between the two ground states, ^**7**^F_**0**_ and ^**7**^F_**1**_ (the lower energy difference between them is approximately 200 cm^**−1**^). The anticipated transitions at 376, 392, 464, 528, 2108, and 2206 nm correspond to ^**7**^F_**0**_ → ^**5**^G_**2**_, ^**7**^F_**0**_ → ^**5**^L_**6**_, ^**7**^F_**0**_ → ^**5**^D_**2**_, ^**7**^F_**0**_ → ^**15**^D_**1**_, ^**7**^F_**0**_ → ^**7**^F_**6**_, and ^**7**^F_**1**_ → ^**7**^F_**6**_ transition [29, 30].

**Fig. 3:**
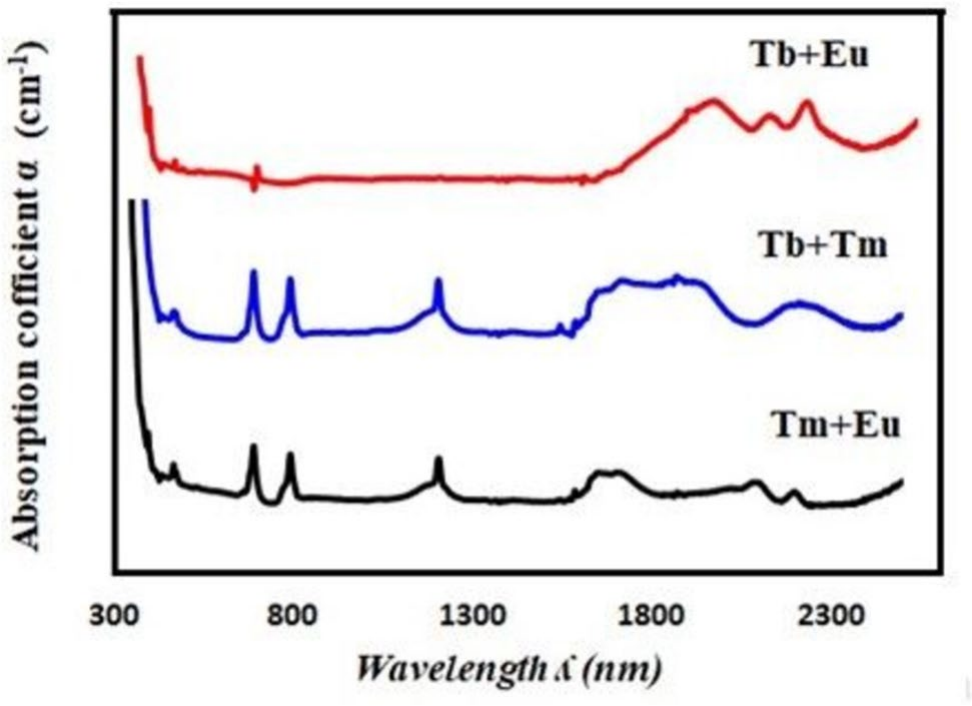
The absorption spectra of the double-rare-earth ion-doped glass samples

**Fig. 4:**
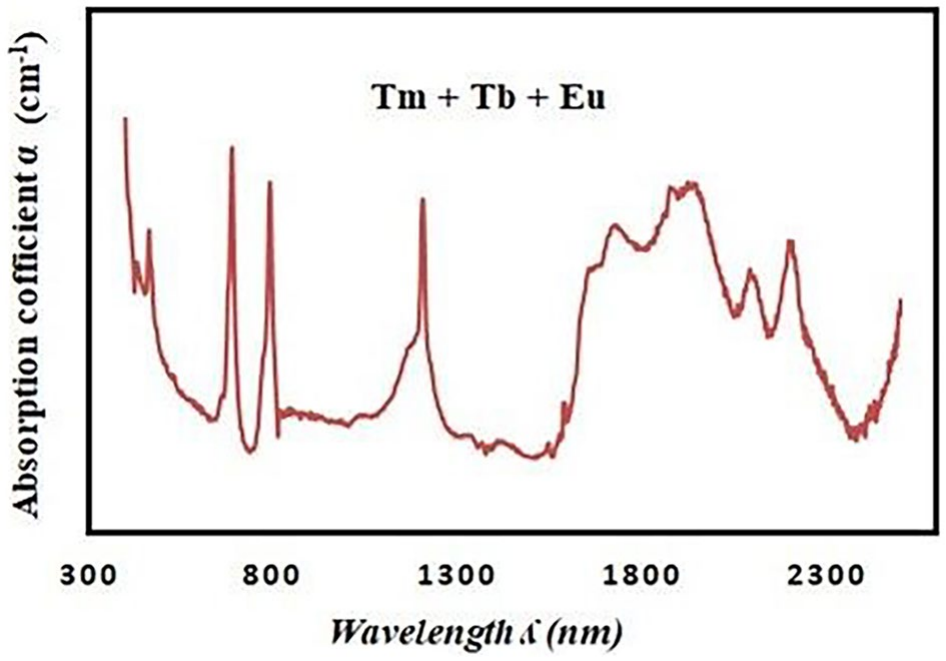
The absorption spectra of the triple-rare-earth ion-doped glass samples

The absorption spectra of the double- and triple-rare-earth ion-doped glass samples are shown in Figs. 3 and 4, respectively. The doped double or triple RE in the glass sample exhibits the appearance of mixing peaks for each RE, as can be seen from these figures.

The absorption spectra of the double rare-earth ion-doped glass samples are shown in Fig. 3. This allowed us to observe the spectra of a material containing Tb^**3+**^ and Eu^**3+**^ ions, which showed five peaks: two in the visible spectrum at 394 and 372 nm and three in the NIR region at 2200, 2100, and 1940 nm. The middle spectrum of the sample containing Tb^**3+**^ and Tm^**3+**^ ions exhibited five peaks: three in the visible region at 790, 685, and 465 nm, two in the near-infrared region at 2200 nm, and a broad peak from 1600 to 2050 nm.

Seven peaks can be seen in the lower spectrum of the sample containing Tm^**3+**^ and Eu^**3+**^: four in the visible spectrum at 790, 685, 460, and 395 nm, three in the NIR region at 2200, 2100, and 1650 nm. The absorption spectra of the triple-rare-earth ion-doped glass samples are shown in Fig. 4. Four peaks in the visible region at 790, 685, 468, and 394 nm, six peaks in the NIR range at 2200, 2100, 1950, 1880, 1700, and 1650 nm are observed in this figure.

**Fig. 5:**
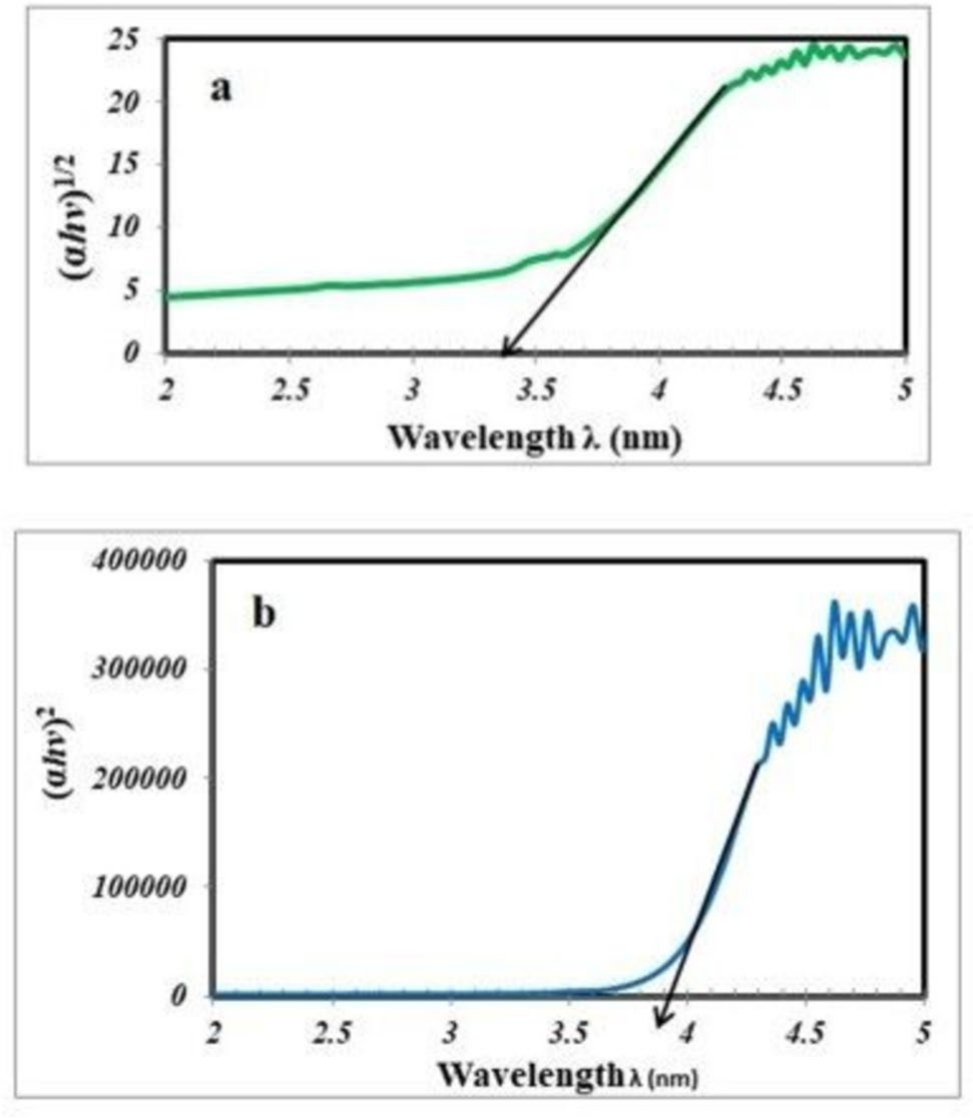
**a** the relationship between (αhν)1/2 and hν **b** the relationship between (αhν)2 and hν

The optical bandgap Eg is a crucial metric for describing the structural and optical characteristics of a material [31] and identifying its type [32]. Semiconductor materials have Eg values between 0 and 4 eV. A substance is an insulator if its value exceeds four. For direct and indirect allowed transitions, Eg is calculated by extrapolating the relationship between the (αhv) power 1/2 or 2 on the y-axis and $hv$ = 0 on the x-axis, as shown by Mott and Davies:

1 $$\alpha ={^{{A(hv- {E}_{g})}^{n}}/_{hv}}$$

where α is the absorption coefficient, n is the type of transition [33], $hv$ is the photon energy, and A is a constant. The Eg values determination are shown in Fig. 5a and b.

Table 2 lists the calculated optical band gap values. Compared with these results, the resulting values largely match the semiconductor property [33–39].

**Table 2:** Lists the calculated optical band gap values

Sample composition mol%			(αhν)^**2**^	(αhν)^**1/2**^
Tm	Tb	Eu	eV	eV
**1**	**---**	**----**	**3.95**	**3.4**
**---**	**1**	**---**	**3.64**	**3.42**
**---**	**---**	**1**	**3.61**	**3.38**
**0.33**	**0.33**	**0.33**	**3.552**	**3.4**
**0.5**	**0.5**	**----**	**3.7**	**3.24**
**---**	**0.5**	**0.5**	**3.7**	**3.26**
**0.5**	**----**	**0.5**	**3.61**	**3.42**

For many different types of photonic devices, the band gap energy is crucial.

The band gap energy has a significant role in determining the emission wavelengths of light emitting diodes and laser diodes. The wavelengths become shorter as the energy level rises. Notably, in conditions with large carrier densities, the highest emission occurs at photon energies that are only a little bit above the band gap energy. This is due to the fact that the density of states drastically increases with frequency.

Only photon energies over the band gap energy can produce a significant response from photodiodes and other semiconductor photodetectors since that is the prerequisite for efficient absorption, which generates the necessary photocurrent. The lower density of states causes the responsivity to typically decline significantly close to the band gap energy.

The host, excitation wavelength, and RE concentration are the three variables that affect the emission of the glass sample.

**Fig. 6:**
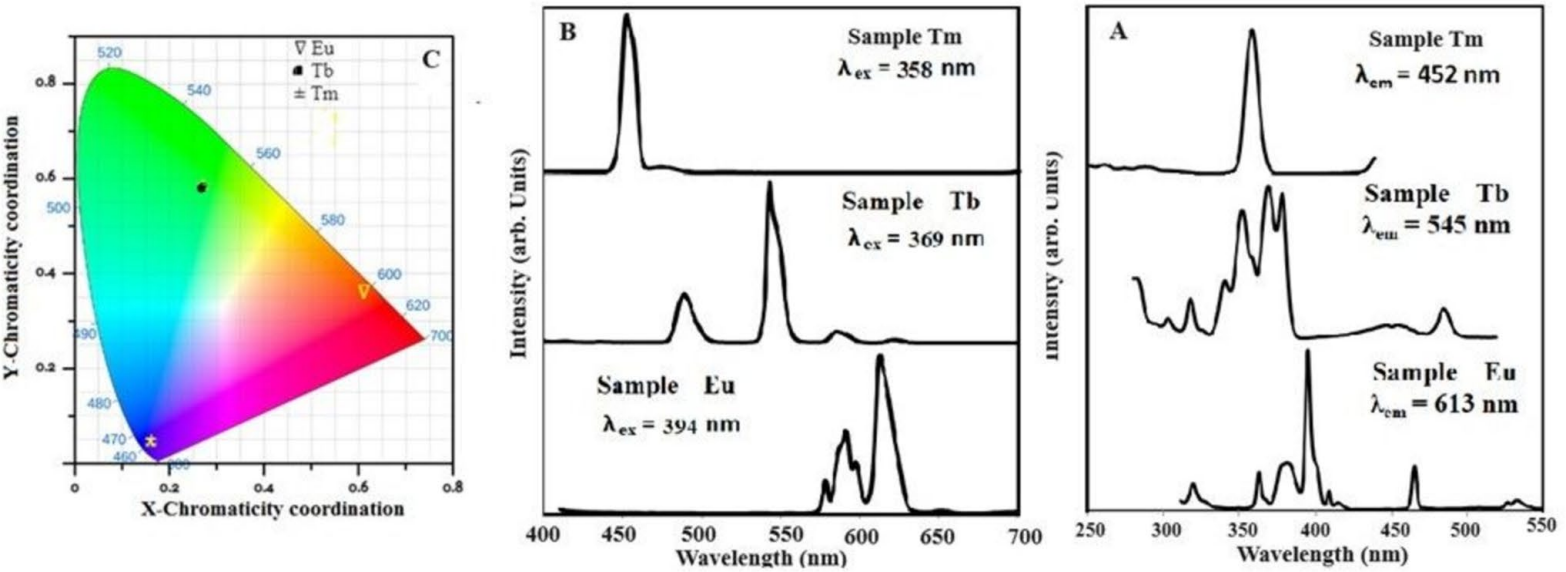
**A** the excitation spectrum. **B** emission spectrum. **C** CIE diagram for a prepared glass sample containing one rare earth oxide

Figure 6A shows the excitation spectrum of the Tm-doped glass sample measured at 452 nm (^**1**^D_**2**_ → ^**3**^F_**4**_). The excitation curve displays a high intensity measured at 358 nm, ascribed to ^**3**^H_**6**_ → ^**1**^D_**2**_, along with low-intensity peaks in the region of 250–300 nm corresponding to ^**3**^H_**6**_ → ^**3**^P_**2**_, ^**3**^P_**1**_, and ^**3**^P_**0**_ + ^**1**^I_**6**_ [23, 40].

The emission spectrum of the sample containing Tm_**2**_O_**3**_ excited at 358 nm is shown in Fig. 6B. According to the emission spectra, the emission peaks at 454 and 476 nm are attributed to transitions between the two ground states at ^3^F_4_ and ^3^H_6_ [22] and are designated as ^**1**^D_**2**_ → ^**3**^F_**4**_ and ^**1**^G_**4**_ → ^**3**^H_**6**_ [23, 40]. Blue emission was linked to a prominent peak at 454 nm [22].

The glass sample containing 1 mol% Tm_**2**_O_**3**_ had chromatic coordinates of x = 0.155 and y = 0.039, which are in the blue region of Fig. 6C and are in accordance with the literature [41].

The monitoring of the emission sample containing Tb^**3+**^ ions at 545 nm is shown in Fig. 6A. Figure 6A depicts the transitions from the ground state of ^**7**^F_**6**_ to ^**5**^H_**6**_, ^**5**^H_**7**_, ^**5**^L_**7**_, ^**5**^L_**9**_, ^**5**^L_**10**_, ^**5**^G_**5**_ + ^**5**^D_**3**_, and ^**5**^D_**4**_ at 300, 317, 340, 352, 369, 378, and 484 nm, respectively [42, 43]. These bands originate from the Tb^**3+**^ ion 4f-4f transitions, and the band at 285 nm causes a ^**4**^F_7_–^**5**^D_1_ transition. The bands at 369, 378, and 352 nm were sufficiently intense to attain the required wavelength to excite the glass sample (Fig. 6B). As shown in Fig. 6B, it was possible to investigate emission at 543 nm (^**5**^D_4_–^**7**^F_5_). This emission has a high intensity and is connected to green emissions ^**5**^D_4_ → ^**7**^F_6_, ^**5**^D_4_ → ^**7**^F_4_, and ^**5**^D_4_ → ^**7**^F_3_ respectively [43, 44]. The chromatic coordinates were x = 0.273 and y = 0.574, which are indicated in the green region in Fig. 6C.

The excitation spectra presented in Fig. 6A are the result of the 613 nm emission observed in the glass sample containing Eu_**2**_O_**3**_. In the excitation spectra, seven peaks were excited from ground states ^**7**^F_**0**_ and ^**7**^F_**1**_ to several excited states [7, 18, 19] with wavelengths of 362, 377, 395, 403, 465, 527, and 534 nm, for transition ^**7**^F_**0**_ → ^**5**^D_**4**_, ^**7**^F_**0**_ → ^**5**^L_**7**_, ^**7**^F_**0**_ → ^**5**^L_**6**_, ^**7**^F_**0**_ → ^**5**^D_**3**_, ^**7**^F_**0**_ → ^**5**^D_**2**_, and ^**7**^F_**0**_ → ^**5**^D_**1**_. In the UV range, high intensity was observed at 395 nm.

Emission spectra of glass sample after excitation at 394 nm are shown in Fig. 6B. Figure 6B shows the creation of an emission peak attributed to Eu^**3+**^ (^**4**^f_**7**_–^**4**^f_**7**_) transitions [45–47] at 579, 592, 613, 657, and 702 nm, corresponding to the ^**5**^D_**0**_ → ^**7**^F_**0**_, ^**5**^D_**0**_ → ^**7**^F_**1**_, ^**5**^D_**0**_ → ^**7**^F_**2**_, ^**5**^D_**0**_ → ^**7**^F_**3**_, and ^**5**^D_**0**_ → ^**7**^F_**4**_ transitions [48]. As a result of how the permitted electric dipole [49, 50] acts when it is influenced by a hypersensitive environment, the peak at 613 nm (^**5**^D_**0**_ → ^**7**^F_**2**_) recorded a high intensity. The peak at 592 nm (^**5**^D_**0**_ → ^**7**^F_**1**_) [50] denotes a magnetic dipole that is permitted, is independent of the local symmetry, and oversees orange emission [42, 51]. As a result, the local environment [52, 53] around Eu equals 1.9, which is higher than unity, describing the Eu^**3+**^ ions in the centric sites [54]. The occurrence of a peak at 657 nm (^**5**^D_**0**_ → ^**7**^F_**3**_) indicates the blending of magnetic and electric characteristics, highlighting the asymmetric location of Eu^3+^ ions in the matrix [42], while also utilizing the emission bands to modulate the total emission color of the glass using the 1931 CIE chromatically [54] theory, as shown in Fig. 6C. From Fig. 6C, it can be observed that the sample's X and Y chromatic coordinates are (0.61, 0.35), which depicts the red–orange light emitted by the glass sample [46, 53, 54].

We can analyze the effect of combining rare earth ions on emission and color owing to differences in color emission with different RE ions. Individual RE ions were studied as described in the previous section.

The two main methods of transferring energy are the multipolar interaction mechanism and the exchange mechanism. The distance between rare earth ions typically serves as the barrier between them. It is the exchange contact mechanism when the critical distance Rc is smaller than 4. When it exceeds 4, the multipole interaction mechanism. There are three different forms of multipole interactions among them: electric dipole–dipole, dipole-quadrupole, and quadrupole–quadrupole [55].

### Tm and Eu Doped Glass Sample

**Fig. 7:**
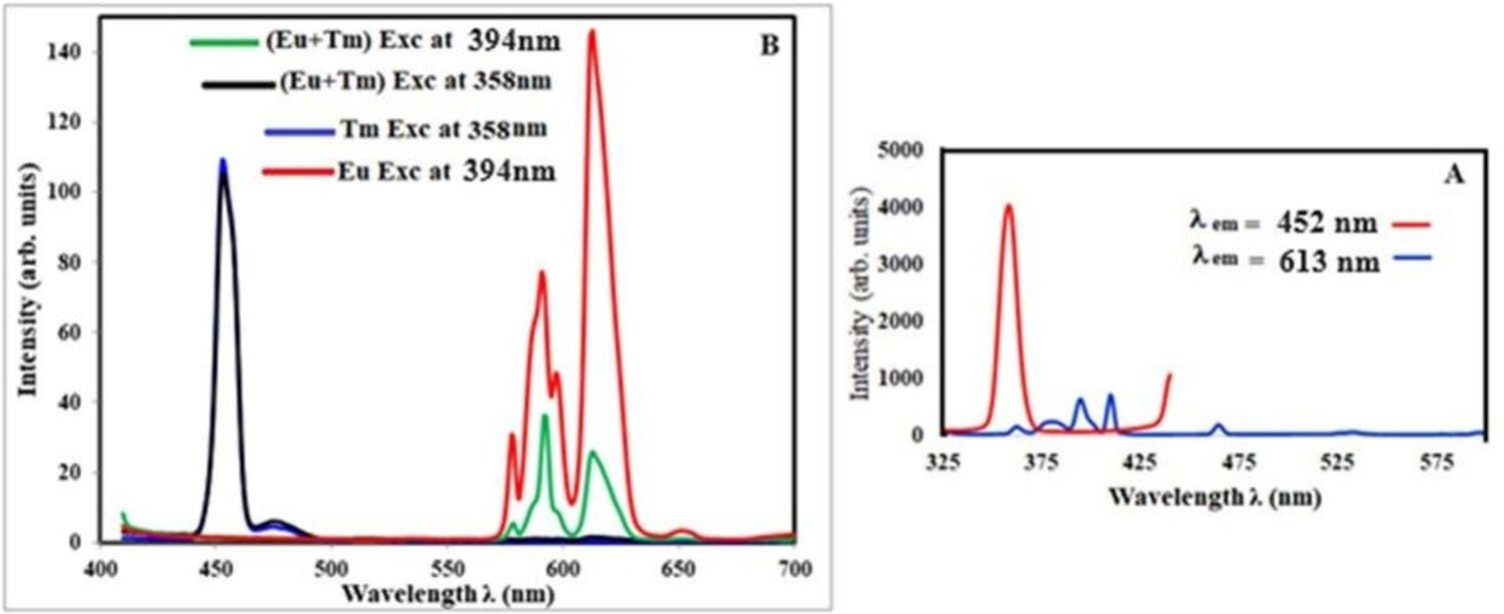
**A** the excitation spectra. **B** the emission spectra of the glass sample containing 0.5 mol% Tm2O3 and 0.5 mol% Eu2O3

Figure 7A shows the excitation spectra of glass samples containing equal amounts of Tm_**2**_O_**3**_ and Eu_**2**_O_**3**_ for measuring the emission at 452 nm and 613 nm.

The distance between Tm^**3+**^ and Eu^**3+**^ (Rc) must first be determined to comprehend the energy transfer between them. This distance was determined using the following formula [56]:

2 $$\mathrm{Rc} = {(\mathrm{M}/\uprho\;\mathrm{Na\;x})}^{1/3}$$

where M is the molecular weight of sample, ρ is density, Na is the Avogadro’s number, x is the concentration of rare earth ions (as shown in Table 3).

**Table 3:** Density and Distance between rare earth ions of glass samples

Sample	Density ρ g/cm^3^	Distance between rare earth ions Rc (A)
Tm	2.354	16.627
Tb	2.376	16.878
Eu	2.307	16.711
Tb + Tm + Eu	2.351	16.721
Tm + Tb	2.365	16.752
Tb + Eu	2.349	16.778
Tm + Eu	2.297	16.749

The values of Rc lead us to believe that the distance between Tm^**3+**^ and Eu^**3+**^ is higher than 4, and that the multipole interaction mechanism is responsible for their transition.

**Fig. 8:**
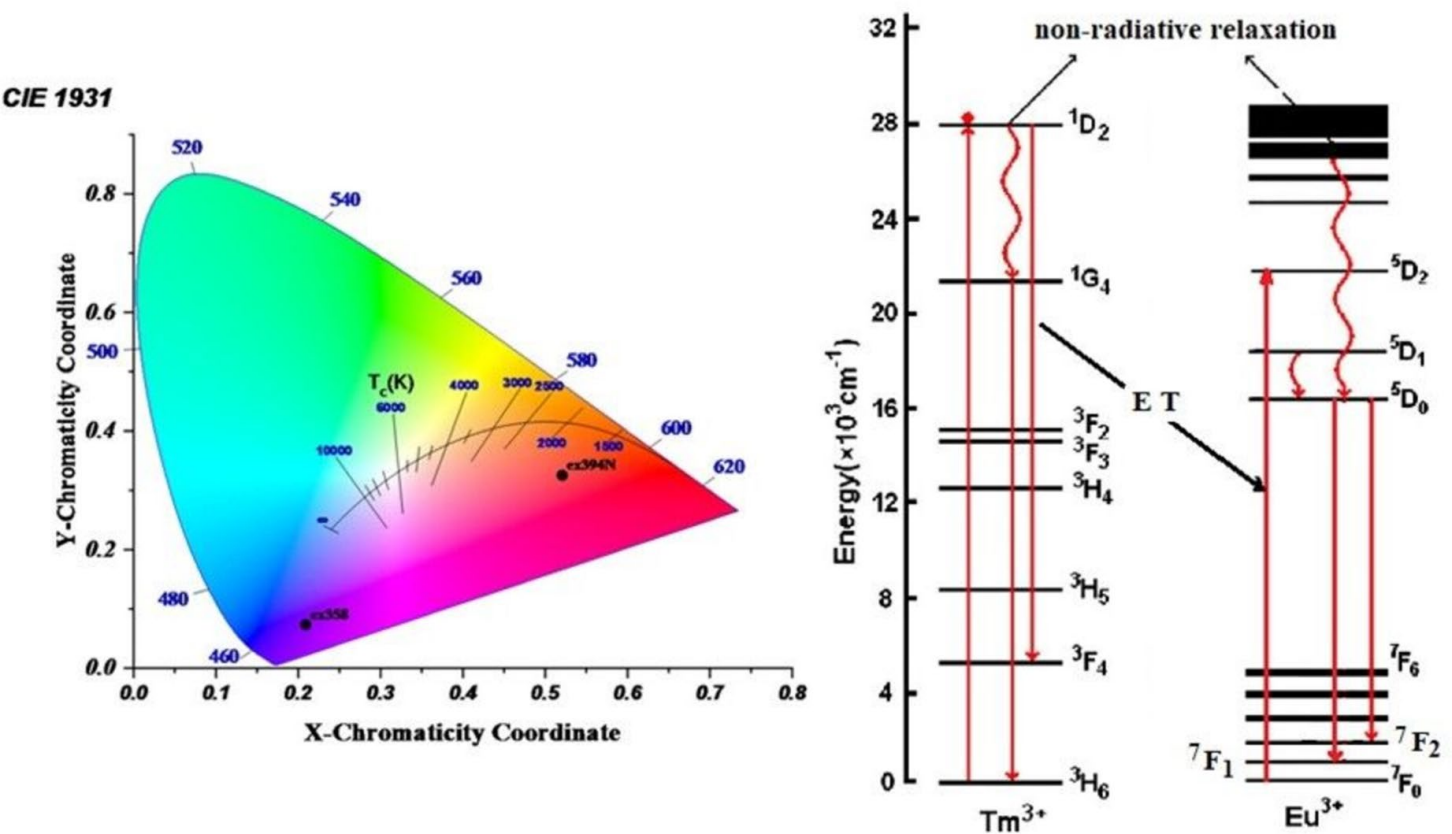
The CIE diagram, and Energy-level diagram of the glass sample containing 0.5 mol% Tm2O3 and 0.5 mol% Eu2O3

Upon sample excitation at 394 nm, the typical emission peaks of Eu^**3+**^ were observed at 591 nm and 613 nm. However, when the sample was excited at 358 nm, emission peaks for both Tm^**3+**^ and Eu^**3+**^ with a weak peak at 613 nm were observed (Fig. 7B). The high emission band at approximately 450 nm, which corresponds to the Tm emission, and the weak band at 613 nm, which corresponds to the Eu^**3+**^ emission, were visible upon excitation at a Tm wavelength of 358 nm. And energy transfer from Tm^**3+**^ to Eu^**3+**^ ions. The examination of the ratio of the varied intensities between ^**5**^D_**0**_ → ^**7**^F_**1,2**_ could determine the symmetry around Eu^**3+**^, even though the Eu excitation wavelength at 394 nm only has the emission peak of Eu^**3+**^. High intensity of ^**5**^D_**0**_ → ^**7**^F_**1**_ over ^**5**^D_**0**_ → ^**7**^F_**2**_. This demonstrates a stronger covalency between Eu^**3+**^ and some oxygen [50] and the fact that the bulk of Eu^**3+**^ ions occupy inversion-symmetry locations [57]. Figure 8 illustrates how the CIE algorithm used the obtained emission data to shift the obtained color toward the center and determined the color chromatically as (0.17, 0.059) for Tm excitation and (0.52, 0.325) for Eu.

The schematic energy level diagram of Tm^3+^ and Eu^3+^ is plotted in Fig. 8. Obviously, the energy gap of Tm^3+1^D_2_ → ^3^F_4_ transitions (approximately 2.18 × 10^4^ cm^−1^) is similar to the excitation gaps of Eu^3+^ (2.15 × 10^4^ cm^−1^) Eu^3+7^F_0_ → ^5^D_2_, which could generate a cross-relaxation process between Tm^3+^ and Eu^3+^ to effectively improve the population of the ^5^D_2_ energy level for Eu^3+^. The energy transfer process involved is as follows:

$${{}^{1}\mathrm{D}}_{2}({\mathrm{Tm}}^{3}+) + {{}^{7}\mathrm{F}}_{0}({\mathrm{Eu}}^{3+})\to {{}^{3}\mathrm{F}}_{4}({\mathrm{Tm}}^{3+}) + {{}^{5}\mathrm{D}}_{2}({\mathrm{Eu}}^{3+})$$

From the above analysis, we can infer the possibility of energy transfer between Tm^3+^ and Eu^3+^. To verify the energy transfer, the fluorescence spectra of the Tm^3+^-Eu^3+^ co-doped host are studied. The emission spectra of the Tm^3+^-Eu^3+^ co-doped host are excited at 358 nm and 394 nm, as displayed in Fig. 7. Under the excitation of 394 nm, the emission spectrum can only observe the characteristic emission peaks of Eu^3+^ at 591 nm and 614 nm. However, the emission peaks of not only Tm^3+^ but also Eu^3+^ can be observed at 358 nm excitation.

### Tm and Tb Doped Host Glass Sample

**Fig. 9:**
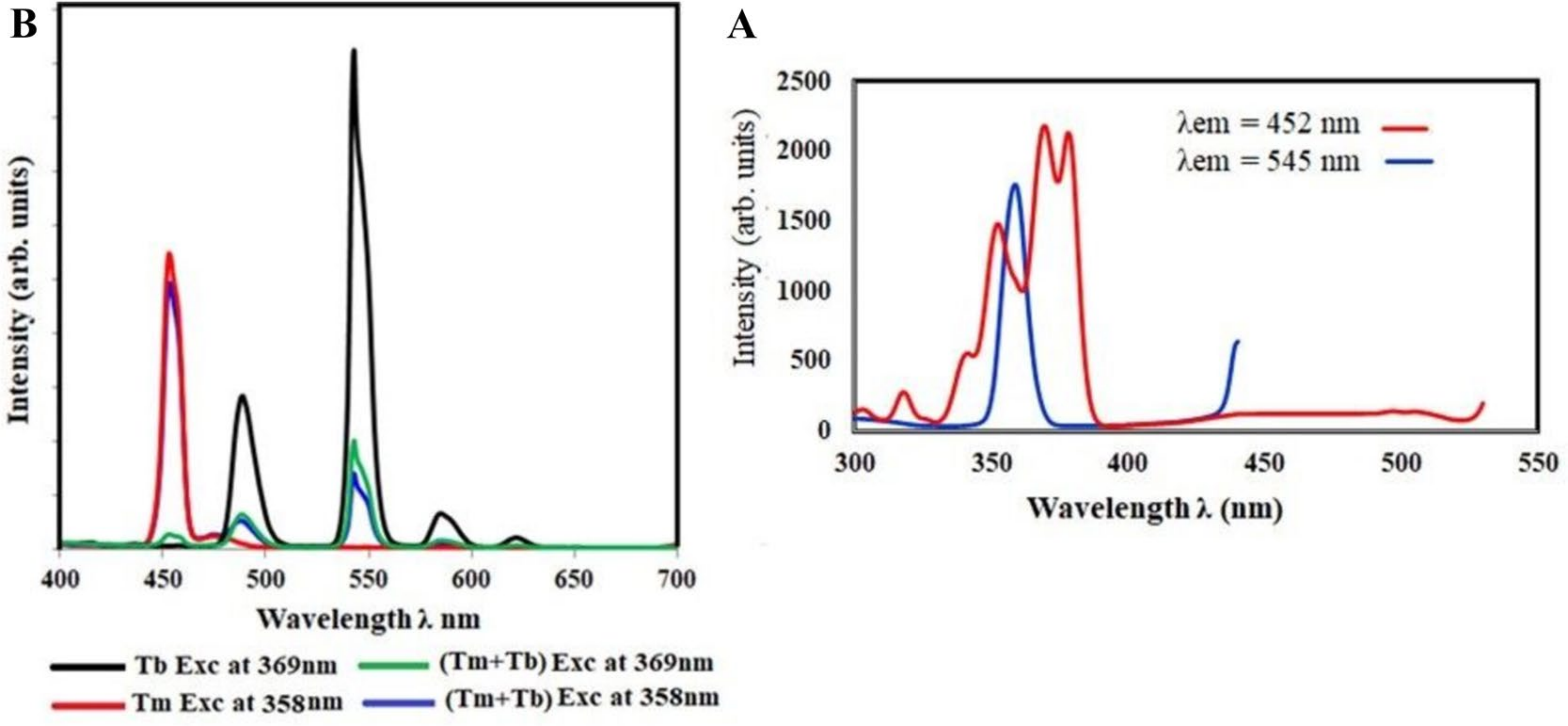
**A** the excitation spectra. **B** the emission spectra of the glass sample containing 0.5 mol% Tm2O3 and 0.5 mol% Tb2O3

The emission spectra of the glass samples had equal amounts of Tm_**2**_O_**3**_ and Tb_**4**_O_**7**_ for measuring the emission at 452 nm and 545 nm, as shown in Fig. 9.

The excitation spectra of the glass sample had an equal amount of Tm_2_O_3_ and Tb_4_O_7_ excited at 369 nm and 358 nm, as shown in Fig. 9.

The values of Rc lead us to believe that the distance between Tm^**3+**^ and Tb^**3+**^ is higher than 4, and that the multipole interaction mechanism is responsible for their transmission.

**Fig. 10:**
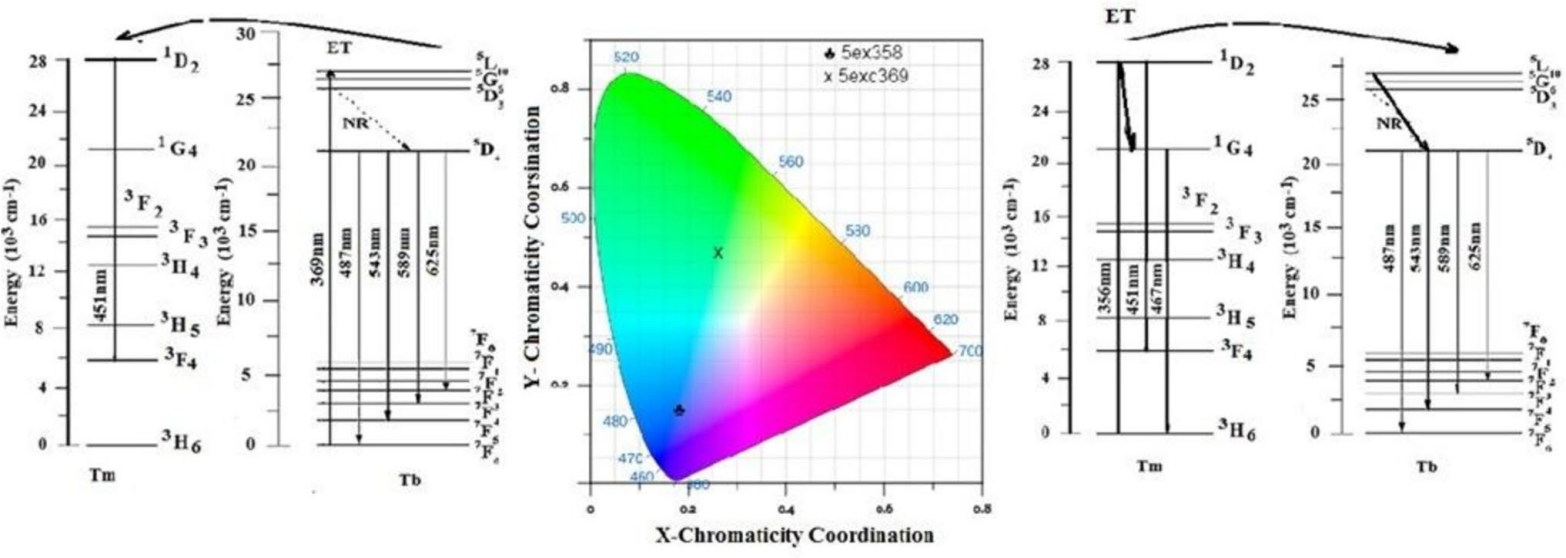
The CIE diagram, and energy level diagram of the glass sample containing 0.5 mol% Tm2O3 and 0.5 mol% Tb2O3

As shown in Fig. 9, when Tb^**3+**^ ions (0.5 mol%) are co-doped with Tm^**3+**^ ions (0.5 mol%) in the host glass, the intensity of the Tm^**3+**^ ion (^**1**^D_**2**_ → ^**3**^F_**4**_) 459 nm emission bands decrease with the addition of Tb^**3+**^ ions. This was attributed to the non-radiative and cross-relaxation mechanisms involved in the energy transfer between the Tm^**3+**^ and Tb^**3+**^ ions. The coincidence of the excitation peaks, as observed in the blending of the two colors (green and blue) from the prior excitation curves for Tm and Tb. In other words, one RE can emit the other RE at the same excitation wavelength. The emission curve was used to observe the emission of the double RE at the same wavelength. In Fig. 9, the excitation at 358 nm is observed, and the ratio of 453 to 543 (the high-intensity peaks of the two RE) is shown to be 1.1, whereas the excitation at 369 nm is shown to be 0.013, showing an energy transfer between the two RE. It is described as having the ability to transfer energy from Tm to Tb [25]. A high-intensity suitable color corresponding to the excitation wavelength was observed. The data analysis from the CIE program (Fig. 10) shows the shifting of the color towards the center.

This could be attributed to the energy transfer among the Tm^**3+**^ and Tb^**3+**^ ions with the following possible non-radiative cross-relaxation channels as shown in Fig. 10: ^**5**^D_**4**_ (Tb^**3+**^) + ^**3**^H_**6**_ (Tm^**3+**^) → ^**7**^F_**0**_ (Tb^**3+**^) + ^**3**^F_**3**_ (Tm^**3+**^) quenches Tb^**3+**^ ions emission bands,

^**1**^D_**2**_ (Tm^**3+**^) + ^**7**^F_**6**_ (Tb^**3+**^) → ^**1**^G_**4**_ (Tm^**3+**^) + ^**7**^F_**0**_ (Tb^**3+**^) quenches Tm^**3+**^ ions emission bands.

### Tb and Eu Doped Host Glass

The values of Rc lead us to believe that the distance between Tb^**3+**^ and Eu^**3+**^ is higher than 4, and that the multipole interaction mechanism is responsible for their transition.

**Fig. 11:**
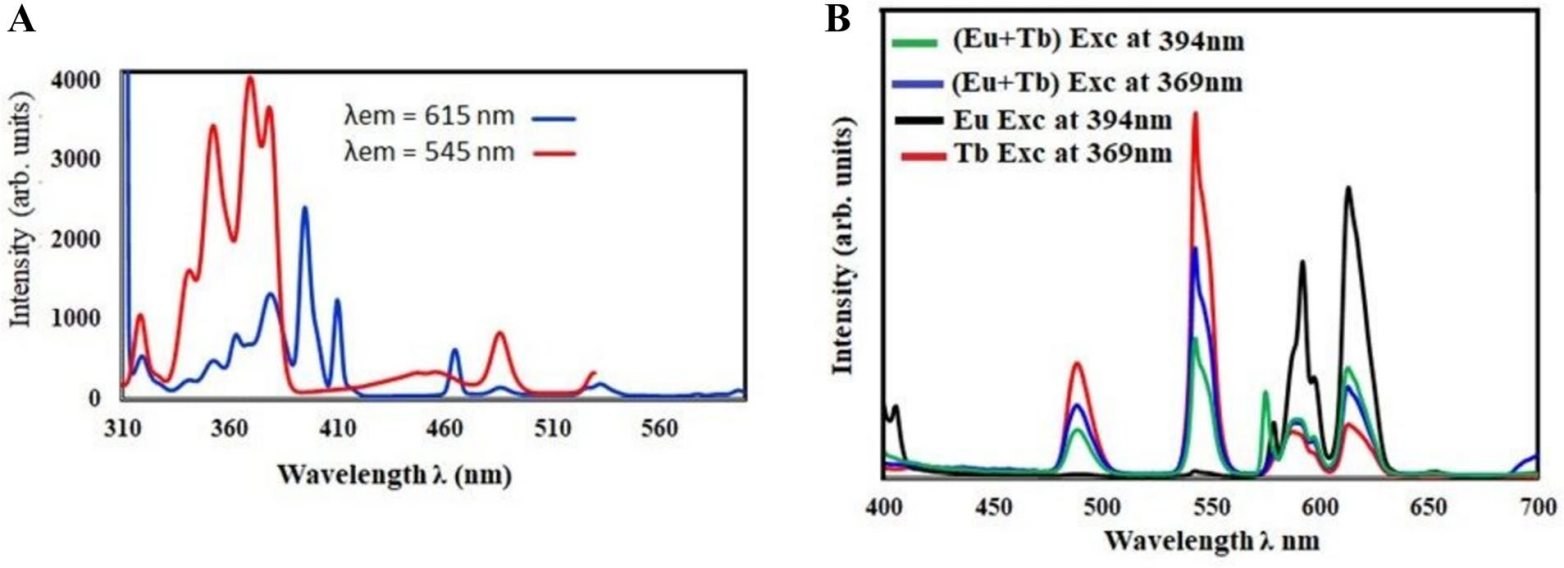
**A** the excitation spectra. **B** the emission spectra of the glass sample containing 0.5 mol% Tm2O3 and 0.5 mol% Eu2O3

Figure 11 shows a comparison of the emission from a glass sample containing equal amounts of Tb_**4**_O_**7**_ and Eu_**2**_O_**3**_ (0.5 mol%) and the emission from the same glass sample containing each element separately (1 mol%) to examine the impact of adding Tb_**4**_O_**7**_ and Eu_**2**_O_**3**_ together on the emission produced from a sample containing one element. The graph shows that Tb^**3+**^-induced luminescence was less intense when Eu^**3+**^ ions were present. The Tb^**3+**^ ions were excited from the ground state to higher excited states, relaxed to the 5D state by multi-phonon relaxation, and finally returned to the ground state (^**5**^D_**4**_ → ^**7**^F_**6, 5, 4**_) of Tb^**3+**^. The energy is either absorbed by Eu^**3+**^ during these transitions and excited to a higher energy level (^**7**^F_**0**_ → ^**5**^D_**1,0**_) or absorbed by Eu^**3+**^ during cross-relaxation, which relaxes non-radiatively to the ^**5**^D_**0**_ level from ^**5**^D_**1**_; finally, a radiative transition occurs from ^**5**^D_**0**_ → ^**7**^F_**J(J=0–6)**_ where the red–orange emission (^**5**^D_**0**_ → ^**7**^F_**2**_) is observed [28]. Figure 11 compares the excitation spectra of the host glass co-doped with Tb^**3+**^ and Eu^**3+**^ to 615 nm of the host glass with only Eu^**3+**^. The co-doped Tb^**3+**^/Eu^**3+**^ excitation spectra revealed a new optical band at 454 nm (^**7**^F_**6**_ → ^**5**^D_**4**_), which was attributed to the absorption of Tb^**3+**^ ions by Eu ions.

**Fig. 12:**
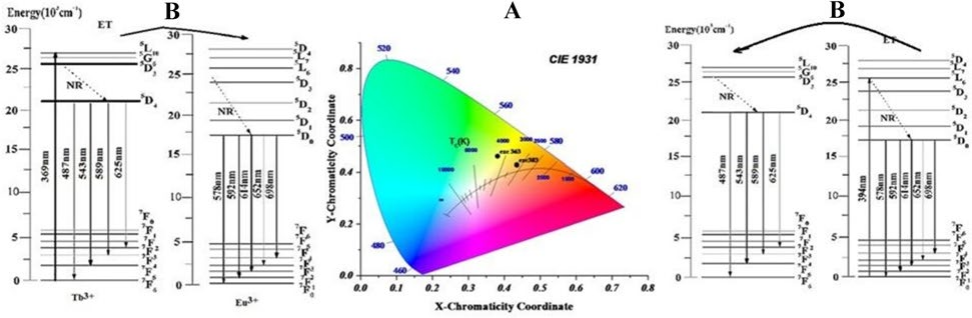
**A** the CIE diagram. **B** energy level diagram of the glass sample containing 0.5 mol% Tm2O3 and 0.5 mol% Eu2O3

Figure 12 shows the CIE diagram of the glass sample containing 0.5 mol% Tb4O7 and 0.5 mol% Eu2O3.

Emission bands of Eu^**3+**^ were measured under excitation of Tb^**3+**^ ions by 543 nm line. The spectrum detected for Tb^**3+**^ singly doped glass consists of four characteristic emission bands, which correspond to ^**5**^D_**4**_ → ^**7**^F_**J (J = 3–6)**_ transitions of trivalent terbium. When a glass sample is co-doped with Tb^**3+**^ and Eu^**3+**^ ions, the additional emission bands are observed. The detailed spectroscopic analysis indicates that these emission bands correspond to ^**5**^D_**0**_ → ^**7**^F_**J (J = 1–4)**_ transitions of Eu^**3+**^. All transitions and energy transfer processes were schematized on the energy level diagrams as shown in Fig. 12.

### Tb, Tm, and Eu Ions Doped Host Sample

**Fig. 13:**
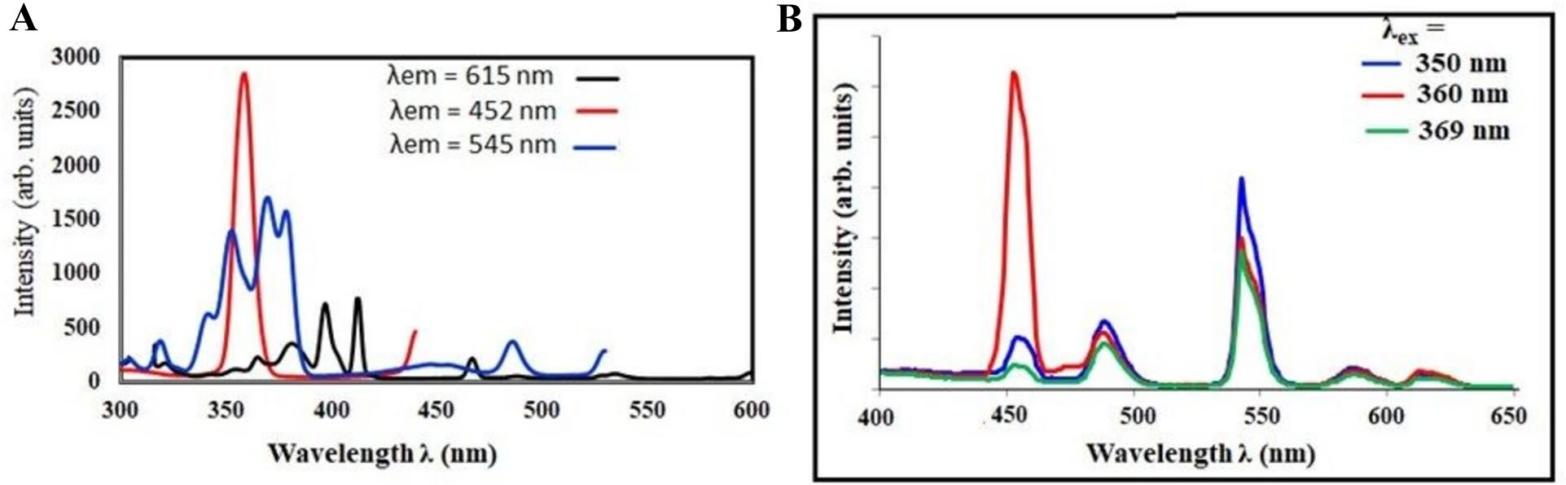
**A** the excitation spectra monitored at 614, 545, and 454 nm for the samples doped with three lanthanide ions, Tm2O3, Eu2O3, and Tb3O7. **B** Emission spectra of a glass sample doped with triple rare-earth ions (Tm/Tb/Eu) at excitation wavelengths of 350, 360, 369, and 395 nm

For the samples doped with three lanthanide ions, Tm_**2**_O_**3**_, Eu_**2**_O_**3**_, and Tb_**4**_O_**7**_, the excitation spectra monitored at 614 nm, 545 nm, and 452 nm are shown in Fig. 13.

In the preceding figures [58, 59], From previous study for sample doped with one rare earth ion Tb or Tm or Eu, the typical excitation bands of Eu^**3+**^, Tb^**3+**^, and Tm^**3+**^ are shown in Fig. 13. Therefore, three excitation peaks were elected to investigate the luminescence effects of Tm^**3+**^, Tb^**3+**^, and Eu^**3+**^. The excitation wavelengths were chosen to be 350, 360, and 370 nm because the excitation spectra shows that there is an overlap between all three excitation peaks from 340 to 385 nm.

Figure 13 shows the emission spectra of a glass sample doped with triple rare-earth ions (Tm, Tb, and Eu) at excitation wavelengths of 350, 360, and 370 nm. This allows the selection of a wavelength within the range of exposure of the glass and displays the ability to emit color in a chromatic diagram.

**Fig. 14:**
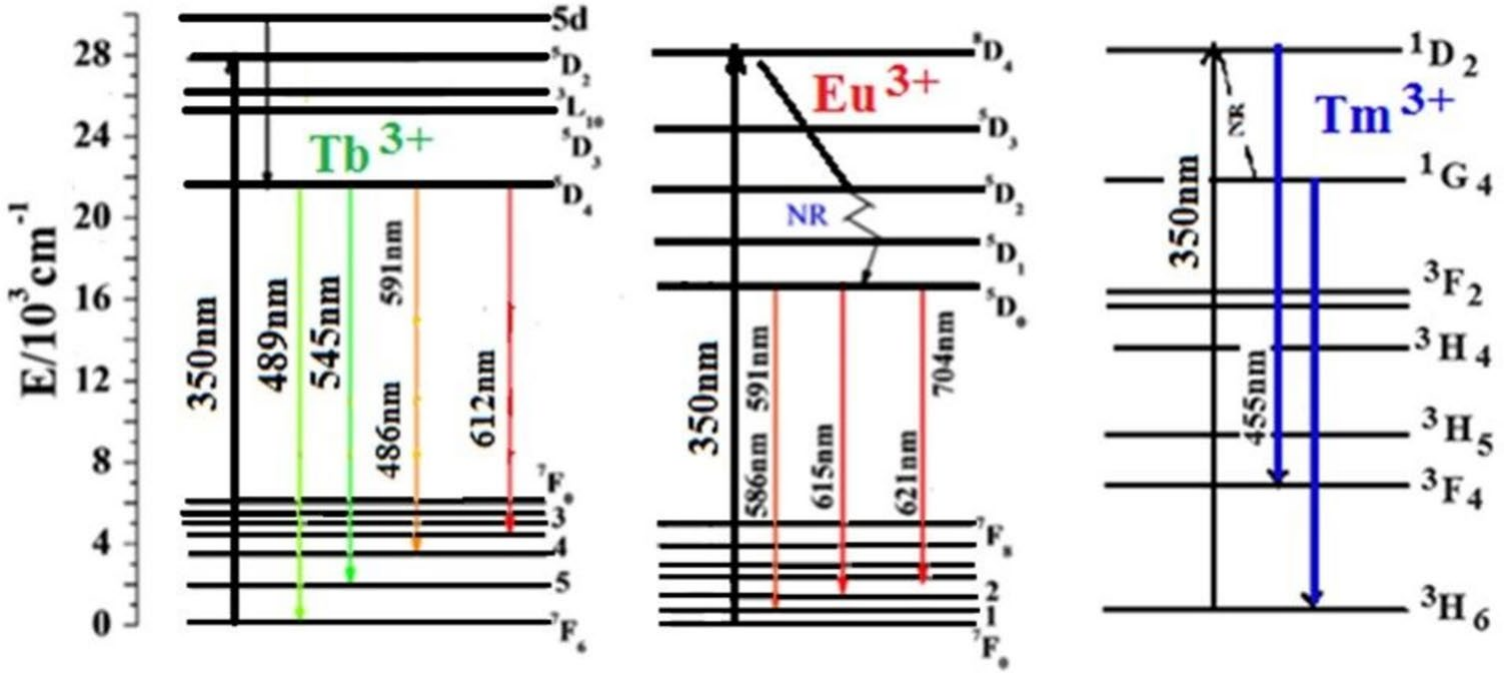
Energy-level diagram and CIE diagram for the prepared glass samples containing equal amounts of Tm2O3, Tb4O7, and Eu2O3

The emission spectra of glass samples with equal mixtures of Eu, Tb, and Tm at various excitation wavelengths (350, 360, and 370 nm) are shown in Fig. 13. Figure 14 shows that energy can be transferred from (Tm^**3+**^ and Tb^**3+**^) to Eu^**3+**^ ions.

Any color determined by the values of triple stimulation X, Y and Z. and related with its hue, saturation, and luminance. The tristimulus values for a non-monochromatic light source with spectral relative power P(λ) are given by:

3 $$\mathrm X=\small {\int}\,\mathrm P(\lambda)\mathrm x(\lambda)\mathrm d\lambda$$

4 $$\mathrm{Y}=\small {\int}\,\mathrm{P}(\lambda )\mathrm{y}(\lambda )\mathrm{d}\lambda$$

5 $$\mathrm{Z}=\small {\int}\,\mathrm{P}(\lambda )\mathrm{z}(\lambda )\mathrm{d}\lambda$$

which gives the power for each of the three primary colors to match with the color of P(λ), and from the tristimulus values the color chromaticity coordinates × and y can be determined using the following expression.

6 $$\mathrm{x}=\mathrm{X}/\mathrm{R}$$

7 $$\mathrm{y}=\mathrm{Y}/\mathrm{R}$$

8 $$\mathrm{z}=\mathrm{Z}/\mathrm{R}$$

where R = X + Y + Z.

The luminescent intensity of the emission spectral measurements can be characterized using the CIE 1931 chromaticity diagram. These chromaticity coordinates are not linearly independent of each other since they follow that × + y + z = 1 for all colors; Third component can always be computed from first two. It is therefore only necessary to quote two of the chromaticity coordinates, and these can of course be plotted on a normal two-dimensional graph [60–62].

**Fig. 15:**
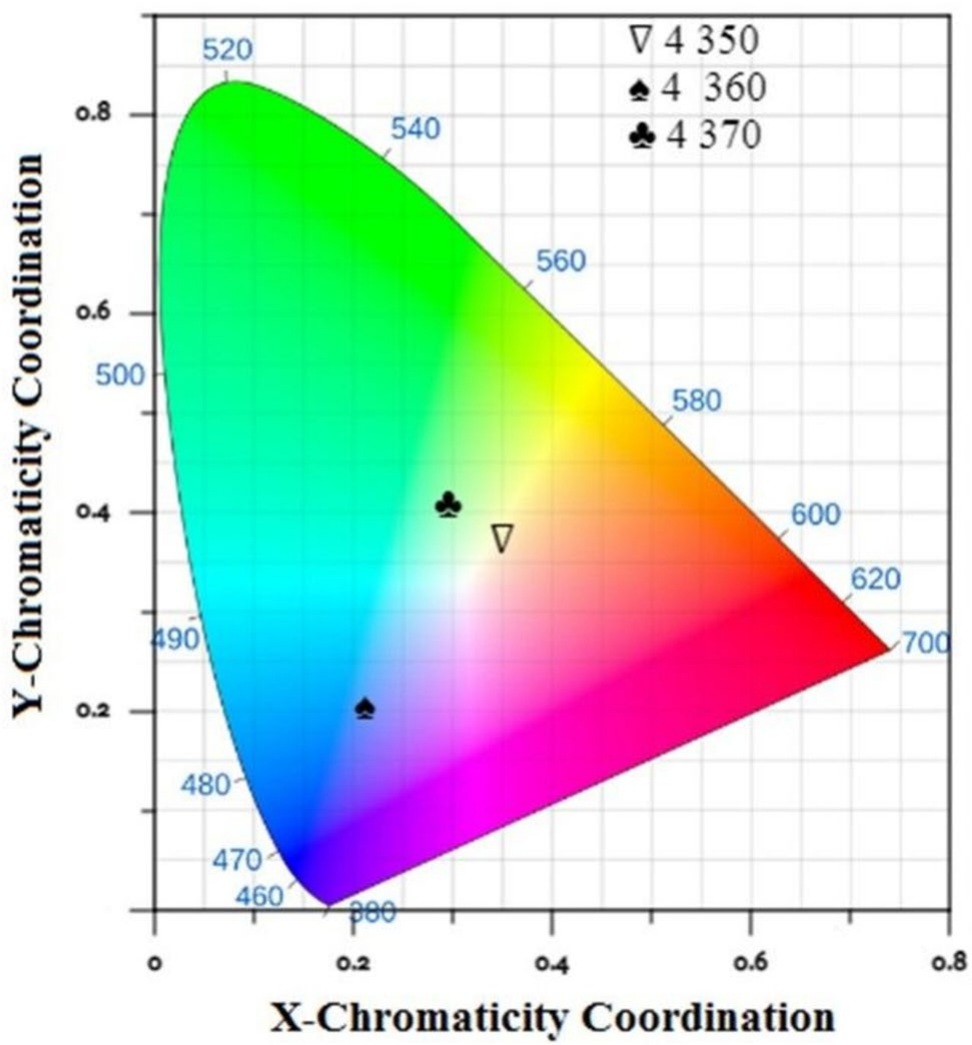
IE diagram for prepared glass sample containing Tm_2_O_3_, Tb_4_O_7_ and Eu_2_O_3_

The color matching function was used to coordinate the emission spectra through CIE 1931 color coordination [63, 64]. Table 4 lists and calculates the coordination of the glass samples with equal concentrations of Tm_**2**_O_**3**_, Tb_**4**_O_**7**_, and Eu_**2**_O_**3**_ excited at 350, 360, and 370 nm. The information acquired explains why certain light emissions appear in different colors. A standpoint (0.345,0.372) in the samples stimulated at 350 nm indicates a white color. At 360 nm, the excited state creates a blue hue, and at 370 nm, a green color, as shown in Fig. 15.

**Table 4:** Lists the excitation wavelength, color coordination, color emission, CCT, and color purity of the glass samples with equal concentrations of Tm2O3, Tb4O7, and Eu2O3 excited at 350, 360, and 370 nm

Sample	λ_ex_ nm	Coordination X, y		Color emission	CCT	Purity Cp
Tm	357	0.155	0.039	Blue	1631	83.94
Tb	369	0.273	0.574	Green	6641	67.49
Eu	395	0.61	0.35	Red orange	1896	83.94
Tm + Tb	358	0.209	0.154	Blue	5875	66.99
	369	0.282	0.461	Green, yellow	6880	37.63
Tm + Eu	358	0.209	0.074	Violet	1684	95.74
	395	0.521	0.326	Red orange	1628	70.94
Tb + Eu	369	0.381	0.461	yellow	4414	37.95
	395	0.435	0.428	Orange	3220	39.46
Tm + Tb + Eu	350	0.349	0.372	White	4922	15.9
	360	0.212	0.205	Blue- white	> 10000	48.62
	369	0.293	0.407	Greenish yellow	6858	21.59

The CCT is an important parameter used in determining the distinctive color produced using McCamy's approximation formula given below [65],

$$\mathrm{CCT}=-449{\mathrm{n}}^{3}+3525{\mathrm{n}}^{2}-6823.3\mathrm{n}+5520.33$$

where n = (x-xe)/(y-ye) is the inverse slope line and xe = 0.332; ye = 0.186.

The type of light bulb used in various regions can be determined using the Kelvin ranges given below.

1. less than 2000 K: emits a soft glow of light, such as candlelight, ideal for dimly lit spaces where ambient illumination is desired.
2. 2000 K-3000 K emits mellow white illumination that frequently appears yellow. Best for living rooms, dining rooms, bedrooms, and outdoor settings.,
3. 3100 K-4500 K: produces strong white light and is ideal for vanities, workstations, kitchens, and other areas where task lighting is required.
4. 4600 K–6500 K emits a bright amount of blue–white light that is similar to that of daylight and is excellent for use in show spaces and work locations. When very strong illumination is required.
5. 6500 K and up to 6500 K Kelvin give off a bright, bluish hue of light often found in commercial locations; they are best for bright task lighting.

The obtained findings displayed the highest values, indicating high brightness. ranging from the white emission that occurs naturally to a cool green emission that is more advantageous for fabricating green solid-state lasers and the green portion of tricolor w-LEDs [66, 67].

## Conclusion

Borate glass was produced with single, double, and triple RE^**3+**^ doping using melt cooling technology. The glass samples under study were activated by the rare earth oxide production Li_**2**_O-Al_**2**_O_**3**_-B_**2**_O_**3**_- RE oxide (RE = Tm_**2**_O_**3**_, Eu_**2**_O_**3**_, and Tb_**4**_O_**7**_), generating orange-red, green, and blue light synchronously when the excited wavelength light affected the samples. Glass samples doped with triple rare earth ions (equal proportion 0.33 mol%) produced white emission when excited by light at a wavelength of 350 nm. verified energy transfer from Tm^**3+**^ and Tb^**3+**^ to Eu^**3+**^. Eu^**3+**^/Tb^**3+**^/Tm^**3+**^ tri-doped glass is a potential candidate for the fabrication of white light-emitting diodes, luminous materials, and fluorescent display devices. There are numerous applications for rare earth luminous materials. They can also be utilized to make medical imaging, radar, computer displays, everyday lighting fixtures, and more in the industrial sector in addition to metallurgy and glass production. The use of rare earth-ion luminous materials in healthcare and the energy sector application. Based on its reversible transmittance and photoluminescence manipulation, rare earth ion-doped transparent glass can be employed as a 3D optical information storage and data encryption medium.

## Data Availability

The data that support the findings of this study are available from the corresponding author upon reasonable request.
